# Validation of blood-based indices associated with sarcopenia for the prediction of pneumonia and delirium in patients with acute withdrawal from excessive alcohol consumption

**DOI:** 10.3389/fpsyt.2026.1759225

**Published:** 2026-01-26

**Authors:** Lanlan Chen, Sha Huang, Xia Lin, Zhouyu Li, Qingrun Chen, Youguo Tan, Xiaoyan Chen

**Affiliations:** The Zigong Affiliated Hospital, Southwest Medical University, Department of Geriatric, Zigong, Sichuan, China

**Keywords:** ALT, AST, blood-based indices associated with sarcopenia, delirium, excessive drinking, pneumonia

## Abstract

**Objective:**

This study aimed to explore the hematology-derived relationship between the blood-based indices associated with sarcopenia including the neutrophil-lymphocyte ratio (NLR), platelet-lymphocyte ratio (PLR) and serum AST/ALT ratio, and the risk of pneumonia and delirium among hospitalized patients experiencing acute withdrawal from excessive alcohol consumption.

**Study design:**

For this retrospective study, patients experiencing acute withdrawal from excessive alcohol consumption who underwent inpatient treatment at a psychiatric teaching hospital in western China between January 1, 2014 and December 31, 2023 were analyzed. Patient-related data were accessed through an electronic medical record database, and logistic regression analyses were used to explore the relationship between the blood-based indices associated with sarcopenia and the risk of developing pneumonia and delirium in this population.

**Result:**

This study enrolled 553 patients. The incidence of pneumonia and delirium in this group of patients was 13.74%. AST/ALT ratios were significantly higher in patients with pneumonia relative to non-pneumonia patients (P<0.001) and in those with delirium versus those with no delirium (P<0.01); compared with the low AST/ALT group, the high AST/ALT group had a higher prevalence of pneumonia (21.90% vs. 11.06%, P<0.01) and delirium (20.44% vs. 11.54%, P<0.05). When the AST/ALT was used as a categorical variable, after adjustment for confounding factors, logistic regression showed that the high AST/ALT group had a higher risk of pneumonia (OR = 1.91, 95%CI: 1.09-3.34) and delirium (OR = 1.92, 95%CI: 1.03-3.58) than the low-AST/ALT group. When the AST/ALT were used as continuous variables, after adjustment for potential risk factors, logistic regression showed that higher AST/ALT was associated with a greater risk of both pneumonia and delirium (pneumonia, OR = 1.48, 95%CI: 1.10-2.00; delirium, OR = 1.58, 95%CI: 1.14-2.19). However, NLR and PLR were not associated with a risk of pneumonia and delirium.

**Conclusion:**

These results suggest that in patients with a history of excessive alcohol consumption and hospitalization for acute alcohol withdrawal, the AST/ALT ratio is associated with increased risk of both pneumonia and delirium, while NLR and PLR are not.

## Introduction

Acute alcohol withdrawal is a serious condition associated with both increased morbidity and mortality, and places a heavy burden on acute healthcare services (1). Pneumonia is one of the major reasons why patients with acute alcohol withdrawal seek medical attention in both outpatient and inpatient settings (2). Our earlier research observed a pneumonia incidence of 13.78% in hospitalized patients with acute alcohol withdrawal syndrome (3). A study by Ahmed et al. found that trauma patients undergoing acute alcohol withdrawal had an approximately three-fold increased incidence of pneumonia (4). Pneumonia is also known to influence the development of delirium tremens in patients with acute alcohol withdrawal (5). Delirium tremens represents the most severe aspect of the condition (1), and patients who develop delirium tremens have longer hospitalization stays and higher total hospital costs compared to non-alcoholics (6). Joy et al. also reported that the presence of delirium tremens in patients with acute coronary syndrome prolonged their hospital stay and increased both their hospital costs and mortality rates (6). In terms of other diseases, patients with sarcopenia have a significantly increased risk of clinical events such as pneumonia and delirium (7, 8). Therefore, it is important to screen for sarcopenia in patients with acute alcohol withdrawal.

Sarcopenia is a disorder of skeletal muscle characterized by marked reductions in muscle mass and strength (9, 10). However, the effective diagnosis of sarcopenia is often difficult in clinical practice as it requires the use of expensive equipment (such as InBody), professional personnel, and patients who are willing and able to cooperate with the testing protocol (3). However, as patients with acute alcohol withdrawal are usually admitted to the hospital due to their reactions to the withdrawal, they may be unwilling or incapable of cooperating in muscle assessments, and thus routine muscle examinations are often not completed at the time of hospital admission.

For these reasons, researchers have attempted to identify biomarkers in the blood that could be used as a more effective and objective tool to screen for sarcopenia. These include the neutrophil-lymphocyte ratio (NLR), platelet-lymphocyte ratio (PLR), and serum AST/ALT ratio, which appear to be particularly promising indicators of the condition (11). Various studies have also shown the NLR to be effective in predicting the risk of both stroke-related and postoperative pneumonia in patients with hip fractures (12, 13), as well as the risk of delirium in older medical patients, and those in intensive care or undergoing esophagectomy (14–16). An elevated PLR is associated with the risk of stroke-related pneumonia and delirium in critically ill patients hospitalized in the intensive care unit (17, 18). The AST/ALT ratio is also predictive of poor prognosis in many other diseases (19–21).

However, there has been no investigation of the use of the above indices in patients with acute alcohol withdrawal. As such, this study was developed with the goal of exploring the application of the blood-based indices associated with sarcopenia (NLR, PLR, AST/ALT) as alternative to muscle testing in these patients, with a particular focus on its relationship with the incidence of pneumonia and delirium in this patient population. This study was based on the hypothesis that the NLR, PLR, AST/ALT can predict the risk of delirium and pneumonia incidence in male patients with a history of excessive alcohol consumption who are hospitalized for acute alcohol withdrawal.

## Methods

### Patient selection

This retrospective study focused on subjects with a history of excessive alcohol intake who received inpatient treatment for acute alcohol withdrawal at a psychiatric teaching hospital in western China from January 1, 2014 to December 31, 2023. Patients experiencing acute withdrawal from excessive alcohol consumption (within 7 days of abstinence from alcohol, with a drinking history of ≥1 year) were included in this study, recording only the first incidence of hospitalization for each patient. The exclusion criteria were female sex, epilepsy, psychosis, malignancy, abstention from alcohol for >7 days before admission, AST or ALT levels >200 U/L (22), alcoholic cirrhosis, missing data on daily alcohol intake, or missing variables included in calculation of the blood-based indices associated with sarcopenia (including neutrophil, lymphocyte, and platelet counts, and AST and ALT levels).

### Ethical oversight

All relevant data were anonymized by the Center for Health Informatics, which reviewed the protocol for this retrospective medical records-based study. Data remained confidential at all times, and this investigation was conducted as per the Declaration of Helsinki. The need for informed patient consent was waived owing to the retrospective design of this study, and the local Research Ethics Committee approved this research effort (No. 202209).

### Excessive alcohol consumption

Excessive alcohol intake was defined as the consumption of more than 25 g per day in males and 15 g per day in females (23). The drinking index was calculated as follows: daily alcohol intake (g/day) * drinking years (alcohol gradus: 52°) (3). Due to the observed high value of the drinking index when used as a continuous variable, it included in the model as a categorical variable. The cohort was divided evenly according to the third quartile (Q3), with the low group representing values less than or equal to Q3 and the high group including values above Q3 (3).

### Blood-based indices associated with sarcopenia

This study only included the results of serum neutrophil, lymphocyte, and platelet counts, as well as AST and ALT levels, from the first collection of venous blood after admission. The neutrophil/lymphocyte, platelet/lymphocyte, and AST/ALT ratios were calculated and evaluated as potential blood-based indices associated with sarcopenia (11). The patients were divided into two groups for each ratio, namely, the high- and low-AST/ALT, high- and low-NLR, and high- and low-PLR groups, according to the Q3 values for the parameters (24).

### Pneumonia diagnosis

Pneumonia was diagnosed by clinicians and treated using appropriate antibiotics. For the present study, only those patients diagnosed with pneumonia for the first time during the follow-up were considered as having pneumonia. Study participants underwent chest radiography or computed tomography (CT), and were considered to have pneumonia if they exhibited acute lung parenchymal inflammation and acute infiltration together with two or more of the following: pyrexia (≥38 °C), hypothermia (<36 °C), chills, sweating, new cough, chest discomfort, changes in discharge color, or difficulty breathing (25).

### Delirium diagnosis

Delirium tremens (hereafter referred to simply as delirium) was diagnosed in accordance with the DSM-5 (26).

### Other data

Patient data were extracted from electronic medical records, including age, duration of drinking, interval from abstinence to hospital admission, smoking history, daily alcohol intake, hypertension, type 2 diabetes, coronary heart disease (CHD), chronic obstructive pulmonary disease (COPD), and blood test results of albumin (ALB), potassium, sodium, and calcium levels after admission.

### Statistical analysis

Data were analyzed using SPSS 25.0 and Python 3.9. Data are reported as means ± standard deviation (SD) when continuous and normally distributed, while medians and interquartile ranges (IQRs) were used to report other continuous data. Numbers and percentages are used to report categorical data. Results were compared with Student’s t-tests, rank-sum tests, Pearson’s chi-square tests, and logistic regression analyses to explore the relationships between the blood-based indices associated with sarcopenia and the risk of pneumonia or delirium tremens in this patient population. These analyses were conducted using either an unadjusted model (Model 1), or a model adjusted for confounding factors with a P-value < 0.05 in univariate analyses (**Tables 1**, **2**). The drinking index was converted into a categorical variable when used as a confounder, separating patients into two groups based on whether values were above or below the median value.

**Table 1:** Characteristics of the study population according to the pneumonia.

General characteristics	Non-pneumonia N=477	Pneumonia N=76	P
Age, year, mean (SD)	52.12(10.15)	55.54(9.21)	**0.006**
Smoking history, n (%)			0.282
no	31(6.55)	2(2.63)	
yes	442(93.45)	74(97.37)	
Drinking index≥10500,n(%)			**0.017**
no	246(51.57)	28(36.84)	
yes	231(48.43)	48(63.16)	
Stop drinking until the time of admission, h, median(iqr)	12(4, 36)	11.5(4, 46)	0.803
COPD, n (%)			**<0.001**
no	464(97.27)	63(82.89)	
yes	13(2.73)	13(17.11)	
Diabetes, n (%)			0.97
no	456(95.6)	72(94.74)	
yes	21(4.4)	4(5.26)	
Hypertension, n (%)			0.93
no	310(64.99)	49(64.47)	
yes	167(35.01)	27(35.53)	
CHD, n (%)			>0.99
no	471(98.74)	76(100)	
yes	6(1.26)	0(0)	
ALB, g/l, mean (SD)	40.42(12.05)	31.28(17.94)	**<0.001**
Electrolyte potassium, mmol/l, n (%)			**<0.001**
normal	389(81.55)	45(59.21)	
abnormal	88(18.45)	31(40.79)	
Electrolyte sodium, mmol/l, n (%)			**0.001**
normal	415(87)	55(72.37)	
abnormal	62(13)	21(27.63)	
Electrolyte calcium, mmol/l, n (%)			0.314
normal	432(90.57)	66(86.84)	
abnormal	45(9.43)	10(13.16)	

COPD, chronic obstructive pulmonary disease; CHD, coronary heart disease; ALB, albumin.

The bold value suggests that the difference is statistically significant.

**Table 2:** Characteristics of the study population according to the delirium.

General characteristics	Non-delirium N=477	Delirium N=76	P
Age, year, mean (SD)	52.82(10.26)	51.16(8.8)	0.138
Smoking history, n (%)			**0.019**
no	24(5.06)	9(12)	
yes	450(94.94)	66(88)	
Drinking index≥10500,n(%)			0.283
no	232(48.64)	42(55.26)	
yes	245(51.36)	34(44.74)	
Stop drinking until the time of admission, h, median(iqr)	10(4, 33)	18(4,58)	0.2
COPD, n (%)			0.157
no	457(95.81)	70(92.11)	
yes	20(4.19)	6(7.89)	
Diabetes, n (%)			>0.99
no	455(95.39)	73(96.05)	
yes	22(4.61)	3(3.95)	
Hypertension, n (%)			0.228
no	305(63.94)	54(71.05)	
yes	172(36.06)	22(28.95)	
CHD, n (%)			>0.99
no	472(98.95)	75(98.68)	
yes	5(1.05)	1(1.32)	
ALB, g/l, mean (SD)	42(8.39)	21.37(22.33)	**<0.001**
Electrolyte potassium, mmol/l, n (%)			**0.022**
normal	382(80.08)	52(68.42)	
abnormal	95(19.92)	24(31.58)	
Electrolyte sodium, mmol/l, n (%)			**<0.001**
normal	416(87.21)	54(71.05)	
abnormal	61(12.79)	22(28.95)	
Electrolyte calcium, mmol/l, n (%)			**0.008**
normal	436(91.4)	62(81.58)	
abnormal	41(8.6)	14(18.42)	

COPD, chronic obstructive pulmonary disease; CHD, coronary heart disease; ALB, albumin.

The bold value suggests that the difference is statistically significant.

## Results

This retrospective study ultimately included 553 patients hospitalized for acute withdrawal from excessive drinking. The incidence of pneumonia and delirium in this group of patients was 13.74%. The average ages of patients with pneumonia and delirium were 55.54 and 51.16 years, respectively, while the average ages of patients without pneumonia and delirium were 52.12 and 52.82 years, respectively.

Compared with patients without pneumonia, those with pneumonia were older. In addition, there were significant differences in drinking index ≥10500, COPD, ALB, electrolyte potassium, and electrolyte sodium levels between the groups (**Table 1**). However, there were no significant differences in terms of smoking history, time from cessation of drinking to admission, diabetes, hypertension, CHD, and electrolyte calcium between the groups (**Table 1**). Significant differences were observed in smoking history, ALB level, electrolyte potassium, electrolyte sodium, and electrolyte calcium between patients with delirium and those without delirium (**Table 2**). However, there were no significant differences in age, drinking index, time from cessation of drinking to admission, COPD, diabetes, hypertension, and CHD (**Table 2**).

**Figure 1:**
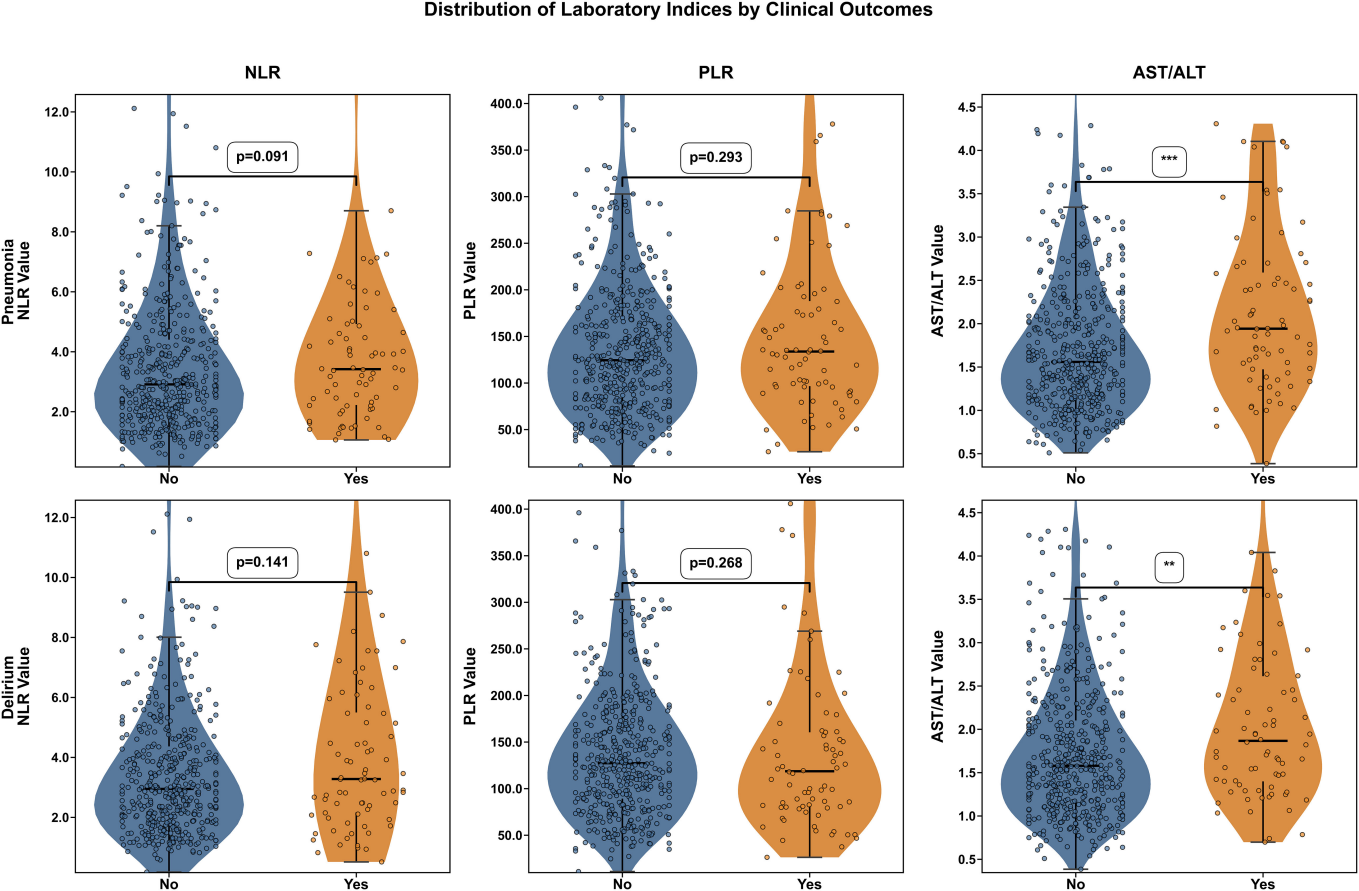
Pneumonia/delirium status and biomarker distribution: NLR, PLR, and AST/ALT. **, represents P < 0.01; ***, represents P < 0.001.

**Figure 2:**
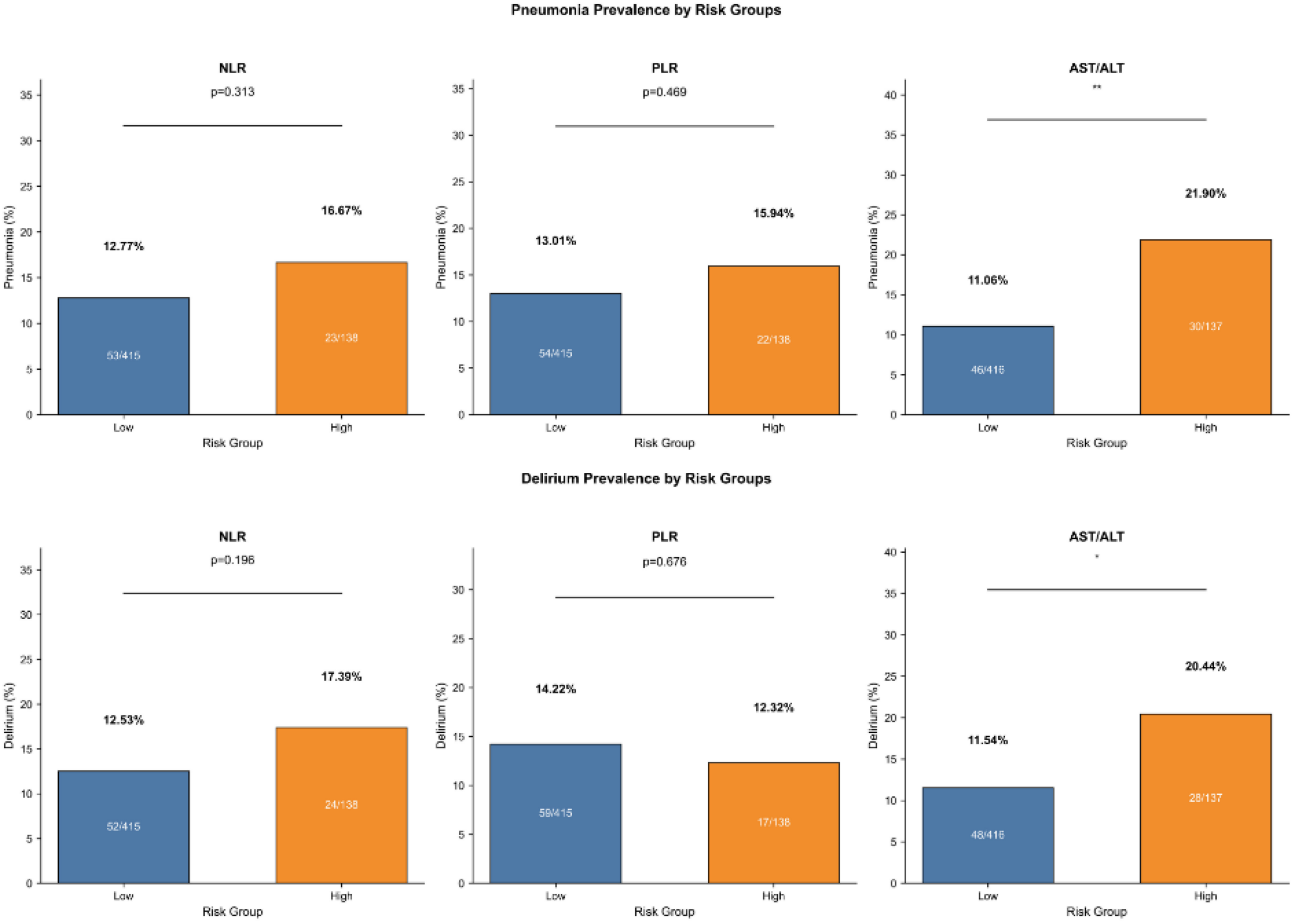
Outcomes prevalence by risk groups of biomarker. *, represents P < 0.05; **, represents P < 0.01.

AST/ALT ratios were found to be significantly higher in patients with pneumonia relative to those without pneumonia (P<0.001) and in patients with delirium compared with those without delirium (P<0.01), as shown in **Figure 1**. However, neither NLR nor PLR values differed between the pneumonia and non-pneumonia groups, nor between the delirium and non-delirium groups. Compared with the low AST/ALT group, the high AST/ALT group had greater prevalence of pneumonia (21.90% vs. 11.06%, P<0.01, **Figure 2**) and delirium (20.44% vs. 11.54%, P<0.05, **Figure 2**). However, there was no difference in the prevalence of pneumonia and delirium between the high- and low-NLR and high- and low-PLR groups.

**Figure 3:**
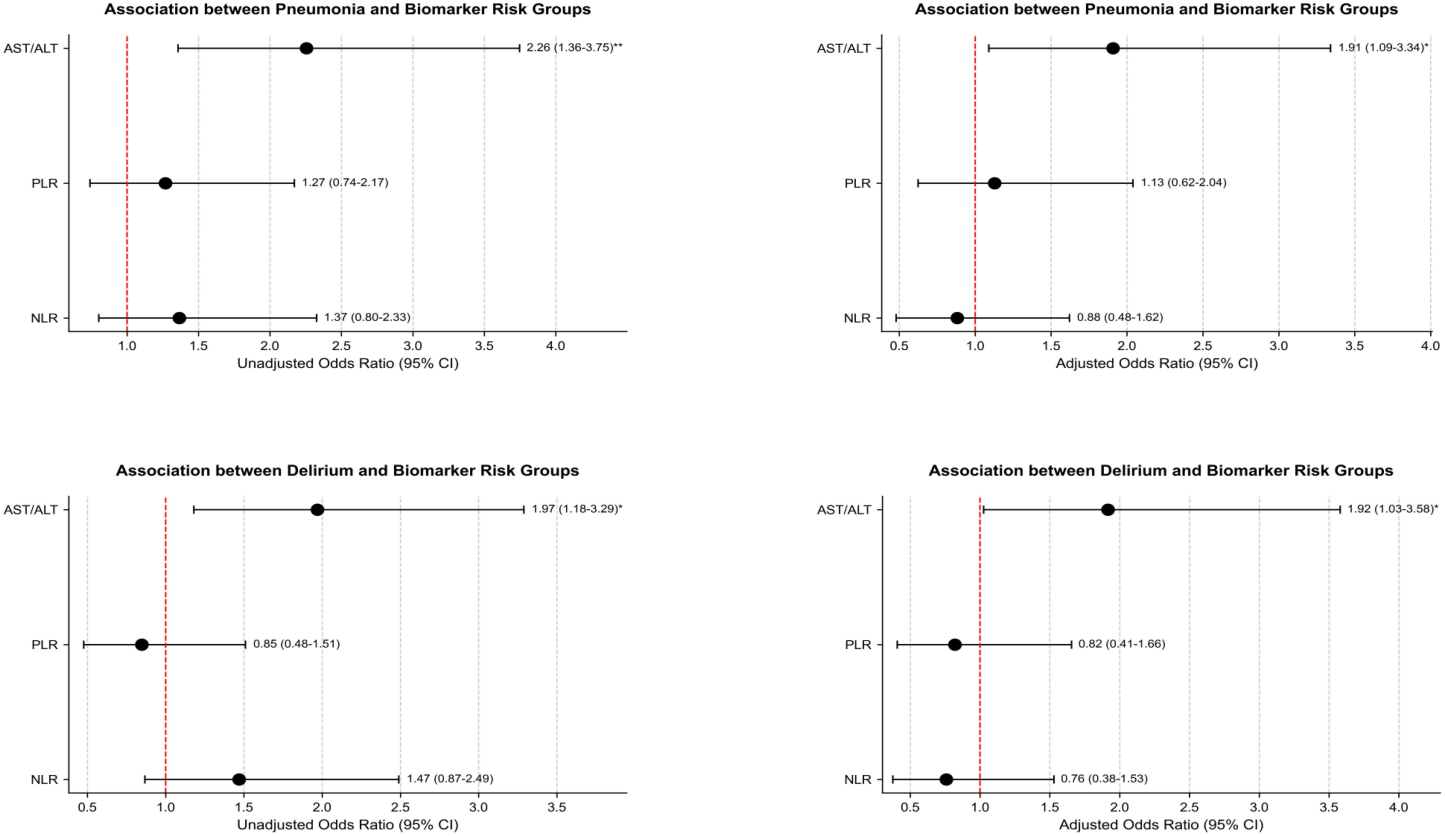
Association between biomarker risk groups and outcomes. Pneumonia, adjusting for age, drinking index, COPD, ALB, electrolyte potassium, electrolyte sodium; Delirium, adjusting for smoking history, ALB, electrolyte potassium, electrolyte sodium, electrolyte calcium.

When the NLR, PLR, and AST/ALT were used as categorical variables, logistic regression analysis showed that patients with high AST/ALT had a higher risk of both pneumonia (OR = 2.26, 95%CI: 1.36-3.75; **Figure 3**) and delirium (OR = 1.97, 95%CI: 1.18-3.29; **Figure 3**) than those with low AST/ALT group. After adjusting for confounding factors, the risk of pneumonia (OR = 1.91, 95%CI: 1.09-3.34; **Figure 3**) and delirium (OR = 1.92, 95%CI: 1.03-3.58; **Figure 3**) in the high AST/ALT group was still higher than that in the low AST/ALT group. However, regardless of adjustment for confounding factors, there were no associations between high and low NLR and high and low PLR with pneumonia and delirium risk.

**Figure 4:**
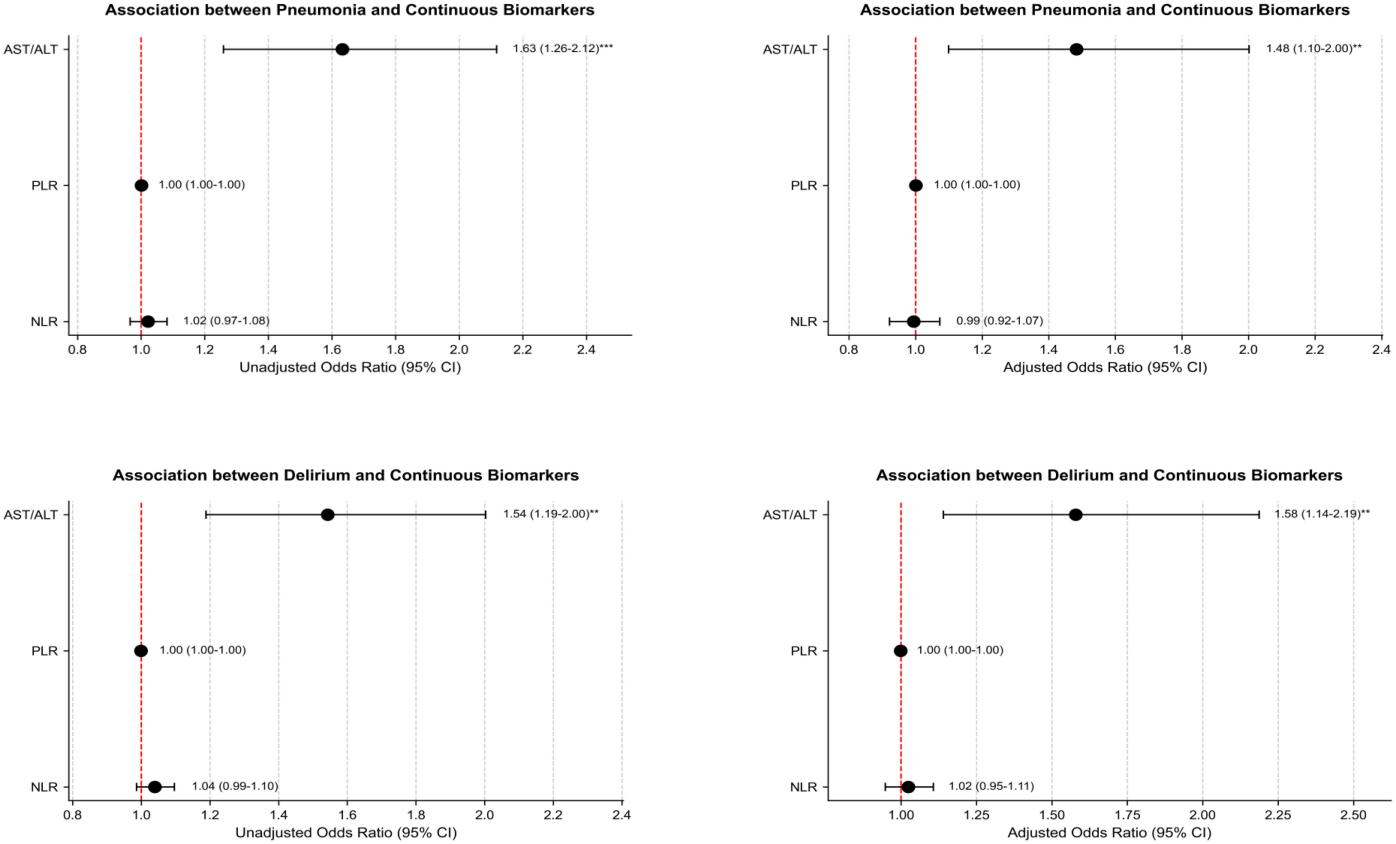
Association between continuous biomarker and outcomes. Pneumonia, adjusting for age, drinking index, COPD, ALB, electrolyte potassium, electrolyte sodium; Delirium, adjusting for smoking history, ALB, electrolyte potassium, electrolyte sodium, electrolyte calcium.

When the NLR, PLR, AST/ALT were used as continuous variables, logistic regression analysis showed that higher AST/ALT values were associated with greater risks of both pneumonia and delirium (pneumonia, OR = 1.63 95%CI: 1.26-2.12; delirium, OR = 1.54, 95%CI: 1.19-2.00; **Figure 4**). After adjusting for potential risk factors, the results still showed that the higher AST/ALT values were linked to increased risk of pneumonia and delirium (pneumonia, OR = 1.48 95%CI: 1.10-2.00; delirium, OR = 1.58, 95%CI: 1.14-2.19; **Figure 4**). However, NLR and PLR were not associated with the risk of either pneumonia or delirium.

## Discussion

The results of this study suggest that blood-based indices associated with sarcopenia (defined by AST/ALT) were significantly associated with the risk of pneumonia and delirium in patients hospitalized for acute withdrawal from excessive alcohol consumption, irrespective of whether AST/ALT was used as a continuous or categorical variable. While previous studies have linked sarcopenia to infections in elderly populations, the present study is the first to demonstrate that a surrogate marker of sarcopenia (AST/ALT) can specifically predict the incidence of complications in patients with acute alcohol withdrawal, a population with a distinct pathophysiology. The use of this index is particularly suitable for psychiatric institutions with patients with acute alcohol withdrawal that are unable to diagnose sarcopenia or in patients who cannot cooperate with sarcopenia examinations, allowing clinicians to predict some adverse outcomes in these patients through simple clinical blood indicators so that appropriate intervention measures can be taken, which will help improve the prognosis of these patients. Furthermore, as a tool for identifying the risk of sarcopenia, the assessment of blood-based indices associated with sarcopenia does not add to the medical costs or radiation exposure of patients. These blood-based indices associated with sarcopenia can also be assessed when the patient is uncooperative, which is particularly advantageous when patients are in a manic state on hospital admission.

Sarcopenia is closely linked with reduced coughing and swallowing functionality (27), both of which are related to the risk of developing pneumonia (27, 28). The skeletal muscle also plays a key role in regulating the function of the immune system, with sarcopenia being associated with the function of immune cell populations including lymphocytes and neutrophils, as well as the production of immunoregulatory cytokines including IL-5, IL-7, and IL-15, all of which are associated with damage (29, 30). Here, a confirmed link between sarcopenia and the risk of delirium was observed among hospitalized for acute alcohol withdrawal following a history of excessive alcohol consumption. Reductions in muscle mass may lead to changes in the volume of distribution and pharmacokinetics for a given drug, leading to a greater risk of drug-induced delirium (31). The pathogenesis of sarcopenia and delirium may also partially overlap, as both groups of patients exhibit elevated serum levels of inflammatory mediators including IL-6 and CRP (30, 32–35). Dysregulated inflammation may thus play a common role in the incidence of delirium and sarcopenia.

The findings showed that use of the AST/ALT ratio as a blood-based indices associated with sarcopenia can predict the risk of pneumonia and delirium in patients with acute withdrawal from excessive drinking. The possible mechanisms involved are as follows: In acute alcohol withdrawal syndrome, there is significant liver damage caused by alcohol, leading to liver dysfunction and metabolic disorders. In addition to reflecting the degree of systemic inflammation, the AST/ALT ratio can also reflect liver damage (36). Although other indicators, such as NLR and PLR, can also reflect systemic inflammation (37), they appear to be unable to capture acute changes resulting from acute liver damage. In acute alcohol withdrawal syndrome, liver damage and dysfunction may also have a direct influence on both systemic inflammation levels and immune function in the patient, thus affecting the development of delirium and pneumonia (38, 39).

The findings of this study show that NLR and PLR were not associated with the risk of pneumonia and delirium in patients with acute alcohol withdrawal and overdose. This is not consistent with previously reported results (40, 41). Several factors may have contributed to this discrepancy. First, studies on this population are generally small-scale and involve substantial heterogeneity. Additionally, differences in laboratory instruments, methods, and reference standards for blood tests in different countries and regions, as well as ethnic variations, may account for these inconsistencies. Second, the average age of the participants in this study was over 50 years, while the average age of those described in the studies by Melamud and Yıldırım was below 50 years. Finally, the criteria and methods used to assess acute alcohol withdrawal and delirium are different. Melamud’s study was based on the ICD-10, while Yıldırım’s study relied on the DSM-5, and our study evaluated acute alcohol withdrawal based on discharge diagnoses with delirium assessed by the DSM-5, which may also have contributed to the differences in findings.

This study has several limitations. First, the number of patients included was relatively small, and the observational and retrospective nature of these analyses may have introduced selection bias, so future larger cohort studies are essential. Second, not all venous blood collected from included patients was fasting venous blood. While some slight changes in blood test indicators were observed after eating, the use of ratios precluded their effects.

## Conclusion

In summary, these results suggest that in patients with a history of excessive alcohol consumption and hospitalization for acute alcohol withdrawal, the AST/ALT ratio, whether viewed as a continuous or categorical variable, is associated with the risk of pneumonia and delirium, while NLR and PLR are not. However, large-scale prospective studies are needed to confirm these conclusions.

## Data Availability

The original contributions presented in the study are included in the article/supplementary material. Further inquiries can be directed to the corresponding authors.
